# The Unfolded Protein Response Protects from Tau Neurotoxicity In Vivo

**DOI:** 10.1371/journal.pone.0013084

**Published:** 2010-09-29

**Authors:** Carin A. Loewen, Mel B. Feany

**Affiliations:** Department of Pathology, Brigham and Women's Hospital, Harvard Medical School, Boston, Massachusetts, United States of America; Boston University School of Medicine, United States of America

## Abstract

The unfolded protein response is a critical system by which the cell handles excess misfolded protein in the secretory pathway. The role of the system in modulating the effects of aggregation prone cytosolic proteins has received less attention. We use genetic reporters to demonstrate activation of the unfolded protein response in a transgenic *Drosophila* model of Alzheimer's disease and related tauopathies. We then use loss of function genetic reagents to support a role for the unfolded protein response in protecting from tau neurotoxicity. Our findings suggest that the unfolded protein response can ameliorate the toxicity of tau in vivo.

## Introduction

Tauopathies are a diverse group of neurodegenerative diseases that are characterized by decline in cognitive and motor function, progressive loss of neurons, and intraneuronal inclusions formed by deposition of the microtubule binding protein tau. Tau is normally unstructured and primarily located in axons. In tauopathies, however, tau becomes hyperphosphorylated, fibrillar, and aggregates in the soma and dendrites of neurons [1].

Alzheimer's disease is the most common tauopathy. Alzheimer's disease is a significant public health concern, being both the most common neurodegenerative disease and the most common cause of dementia. In addition to intracellular tau inclusions, Alzheimer's disease brains also contain extracellular aggregates of amyloid β (Aβ) protein, derived via proteolysis from the larger transmembrane amyloid precursor protein (APP). How Aβ and tau contribute to the pathology of Alzheimer's disease is still unclear; however, the appearance and anatomical distribution of tau inclusions in Alzheimer's disease brains correlates well with neuronal loss, suggesting that tau plays an important role in the progression of the disease [2]. Indeed, mutations in tau have been found in patients with the inherited tauopathy known as frontotemporal dementia with Parkinsonism linked to chromosome 17 (FTDP-17) [3]–[5]. These mutations establish that tau dysfunction can cause neurodegeneration and dementia. Thus, determining the molecular pathways that modify tau-mediated neurotoxicity will better our understanding of the pathology of all tauopathies, including Alzheimer's disease, and may uncover potential therapeutic targets.

One candidate for such a pathway is the unfolded protein response (UPR) because the UPR appears to be upregulated in Alzheimer's disease [6], [7]. The UPR is activated by endoplasmic reticulum (ER) stress [8], [9]. The ER is a cellular organelle with a variety of functions, including the synthesis of lipids, carbohydrates, transmembrane proteins and proteins destined for secretion or transport to other cellular organelles. Newly synthesized proteins in the ER lumen are properly folded there, which may include the formation of disulfide bonds. Many post-translational modifications also occur in the ER lumen. The function of the ER can be compromised by a variety of stressors, including inhibition of disulfide bonding, disruption of glycosylation, depletion of ER calcium, blocking ER to Gogi transport, increasing the ER's protein synthesis load, accumulation of aberrant proteins that do not fold properly, and disruption of ER associated protein degradation (ERAD). Many of these insults result in an increased concentration of mis- or unfolded proteins in the ER. Upon ER stress, sensors in the ER lumen that can detect mis- or un-folded proteins start the intracellular signal transduction pathway called the UPR. The first aim of the UPR is to restore ER homeostasis by decreasing the protein load that enters the ER, stimulating the degradation of accumulated misfolded proteins, up-regulating the expression of genes that function in ER protein folding, and expanding the size, and thus capacity, of the ER. However, if homeostasis cannot be achieved and the UPR is sustained, the UPR can also trigger cell death. The molecular mechanisms that mediate the death of ER-stressed cells are less well understood than those that mediate the restoration of ER homeostasis [9].

The ER stress sensors in the ER lumen activate three proteins located in the ER membrane: inositol-requiring protein-1 (IRE-1), activating transcription factor-6 (ATF6) and protein kinase RNA-like ER kinase (PERK). IRE-1 is a ser/thr kinase with endonuclease activity. When activated, it catalyzes the splicing of Xbp1 mRNA, which causes a frame shift during Xbp1 translation. The spliced Xbp1 protein is a transcription factor that contains a bZIP domain as well as a potent trans-activation domain. It up-regulates a variety of UPR-related genes involved in protein entry to the ER, protein folding, ERAD, and protein quality control. Activated IRE-1 also binds the adaptor protein TRAF2 to activate the apoptosis signal-regulating kinase 1 (ASK1) and cJun-N terminal kinase (JNK) pathway. ATF6 is also a bZIP transcription factor that, when activated by ER stress, controls the expression of many UPR genes related to ERAD, protein folding and protein quality control in the ER. Activated PERK inhibits global protein translation, thereby reducing ER load, by phosphorylating and inactivating eIF2α. PERK mediated phosphorylation of eIF2α also leads to the translation of ATF4, a transcription factor that induces the expression of genes that function in amino acid metabolism, the antioxidant response and apoptosis regulation [9].

Examination of postmortem material from patients with Alzheimer's disease has supported activation of the UPR [6], [7]. Given the known role of the UPR in misfolding in the secretory pathway, much work has focused on the role of APP, the presenilins and the small Aβ peptides derived from APP in influencing the activity of the UPR [9]. Indeed, cell culture studies demonstrate that Aβ peptides can activate the UPR [10], [11]. In contrast, Unterberger et al. [7] found that activation of the PERK-eIF2α pathway was not correlated with extracellular deposits of Aβ. Other studies have, in fact, suggested a relationship between tau phosphorylation and activation of the UPR [12]. These latter findings implicate tau in activation of the UPR in Alzheimer's disease, although Aβ might still be playing a role either through indirect action or soluble oligomeric species.

To address the role of tau in activating the UPR we have used an in vivo genetic model of tauopathy in *Drosophila*. Expression of human tau in *Drosophila* neurons recapitulates many important aspects of human tauopathy, including age-dependent neurodegeneration [13], phosphorylation of tau [14]–[17], formation of actin-rich cytoplasmic inclusions [18], oxidative stress [19], and cell cycle-dependent apoptotic neuronal death [20]. We utilized our *Drosophila* tauopathy model to explore the cause and significance of UPR activation in tau-mediated neurodegeneration. We found that the UPR is activated in a phosphorylation-dependent fashion in tau transgenic flies, and that UPR activation protects from tau-mediated neurotoxicity.

## Results

**Figure 1:**
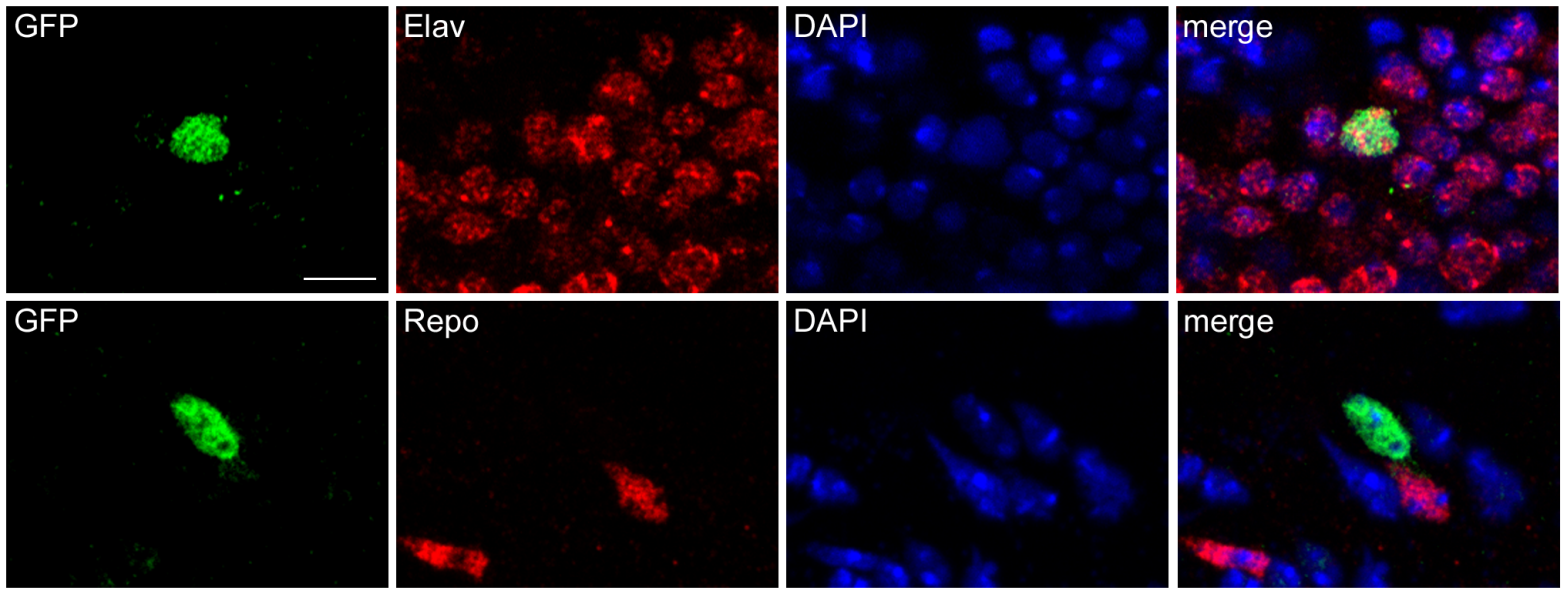
Upregulation of unfolded protein response. Splicing of an Xbp1-EGFP reporter construct in neurons (marked by anti-elav immunostaining, upper panels), but not glia (marked by anti-repo immunostaining, lower panels) in flies expressing tau^R40W^ using the panneural *elav-GAL4* driver. Scale bar is 5 µm. Flies are 10 days old.

The tauopathy model used in the current studies is based on expression of human tau in *Drosophila*. In our model the bipartite UAS/GAL4 expression system [21] is used to express human tau in a panneural pattern with the *elav-GAL4* driver. The model has been previously described in detail [13]–[20]. To determine if the UPR is activated in our tauopathy model we utilized a *UAS-Xbp1-EGFP* reporter system developed by Ryoo et al. [22]. In the reporter construct, EGFP is located after the IRE-1 splice site in Xbp1 such that EGFP is only in frame after Xbp1 has been spliced by IRE-1. Thus the UAS-Xbp1-EGFP transgene acts as a reporter for UPR activation as expression of EGFP indicates that Xbp1 has been cleaved by IRE-1. The *UAS-Xbp1-EFGP* transgene was first co-expressed with human tau in *Drosophila* neurons using the *elav-GAL4* driver. The FTDP-17 associated mutant version of tau, tau^R406W^, was used in these experiments because expression of mutant tau^R406W^ induces a level of toxicity well suited for experimental analyses [18]–[20]. We observed activation of the UPR reporter in adult transgenic tau animals (Figure 1). Activation was apparent throughout the brain, consistent with the panneural pattern of the *elav-GAL4* driver and the widespread neurodegeneration present in tau transgenic flies [13]. To determine the cell type with reporter activation, we double stained for elav, a marker of neurons, or repo, a glial marker. We observed activation of the reporter in neurons (Figure 1, upper panels), but not glia (Figure 1, lower panels). To confirm activation of the UPR in tau transgenic flies we used an enhancer trap P element inserted into the *Hsc70-3* locus. We found significant activation of Hsc70-3 in tau transgenic flies (27.6±2.6 ß-galactosidase immunoreactive cells per brain; genotype: *elav-GAL4/Hsc70-3^G0407^;UAS-tau^R406W^/+*) compared to controls (1.2±0.5 ß-galactosidase immunoreactive cells per brain; genotype: *elav-GAL4/Hsc70-3^G0407^*) at 10 days of age.

**Figure 2:**
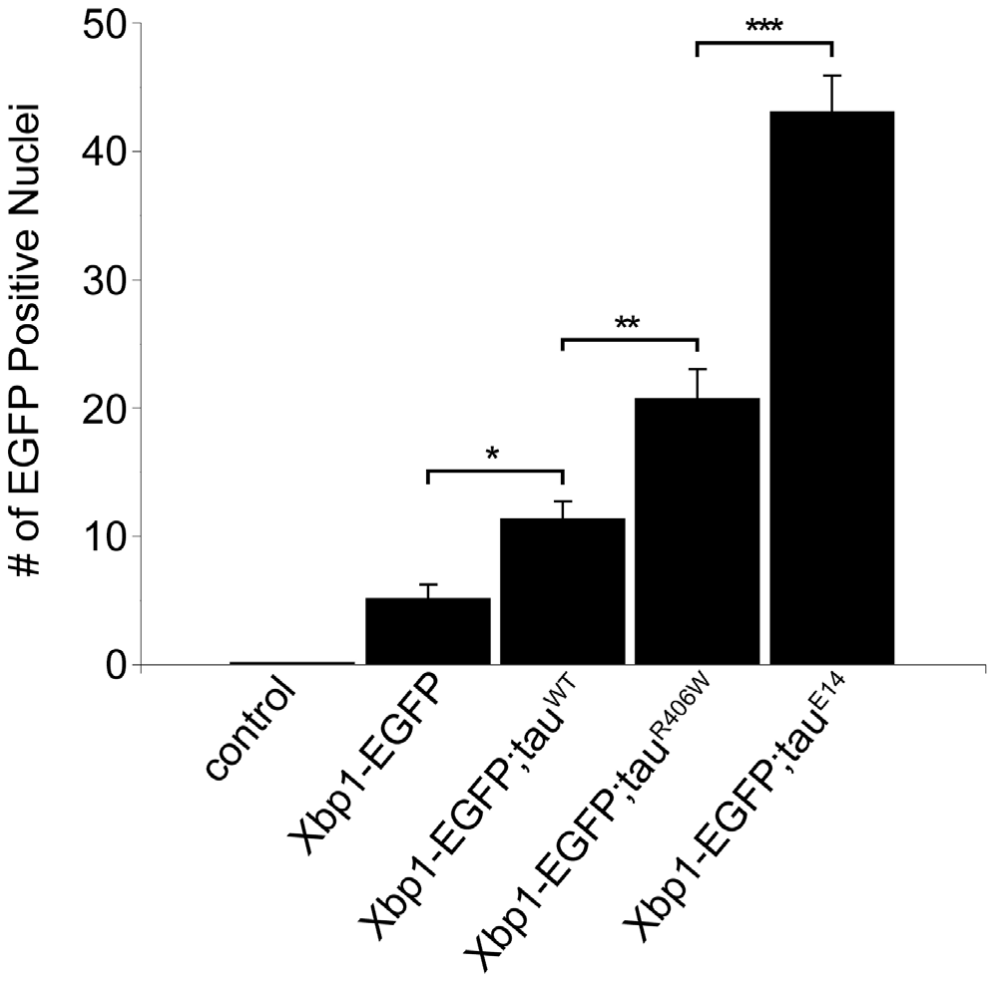
Quantitative analysis of Xbp1-EGFP reporter activity. Significant activation in tau transgenic flies, with the degree of activation correlating with the degree of toxicity of various forms of human tau, including wild-type tau (tau^WT^), FTDP-17 linked mutant tau^R406W^ and the phosphomimic mutant tau^E14^. No immunoreactivity for GFP in control animals (*elav*-*GAL4*/+). *P<0.05, **P<0.01, *** P<0.001, one-way ANOVA with Tukey test for multiple comparisons. Flies are 10 days old.

To further the explore the relationship between tau expression and UPR activation we quantified the degree of UPR activation flies expressing tau transgenes that cause varying levels of neurotoxicity. At 10 days of age, expression of human wild-type tau (tau^WT^) in *Drosophila* neurons is mildly toxic, expression of the FTDP-17 linked mutant tau (tau^R406W^) is moderately toxic, and expression of a phosphomimetic mutant tau in which 14 disease-associated SP/TP phosphorylation sites are mutated to glutamate (tau^E14^) is substantially more toxic [16]–[20]. Western blot analysis confirmed that differences in neurotoxicity were not due to different levels of tau expression (Figure S1), consistent with prior characterization of these mutant lines [13], [19], [20]. For quantitative analysis, the number of EGFP positive nuclei was counted in 4 µm serial sections of entire fly brains. UPR activation was absent in control flies and minimal in flies expressing only the *UAS-Xbp1-EGFP* reporter at 10 days of age (Figure 2), or at one day or 30 days of age (data not shown). In contrast, activation of the reporter was robust in flies expressing all forms of tau (Figure 2). The degree of UPR activation correlated very well with the degree of toxicity caused by the various forms of tau (tau^WT^ = 11.3±1.4, tau^R406W^ = 20.7±2.4, tau^E14^ = 43.0±2.9). Activation of the UPR in tau transgenic flies was age dependent (tau^R406W^ = 6.7±1.1 at one day of age), consistent with our prior characterization of age-dependent neurodegeneration in tau transgenic flies [13]. Our findings suggest a plausible link between tau neurotoxicity and UPR activation. Further, the substantially increased UPR activation seen with expression of pseudophosphorylated tau supports a role for tau phosphorylation upstream of UPR activation.

**Figure 3:**
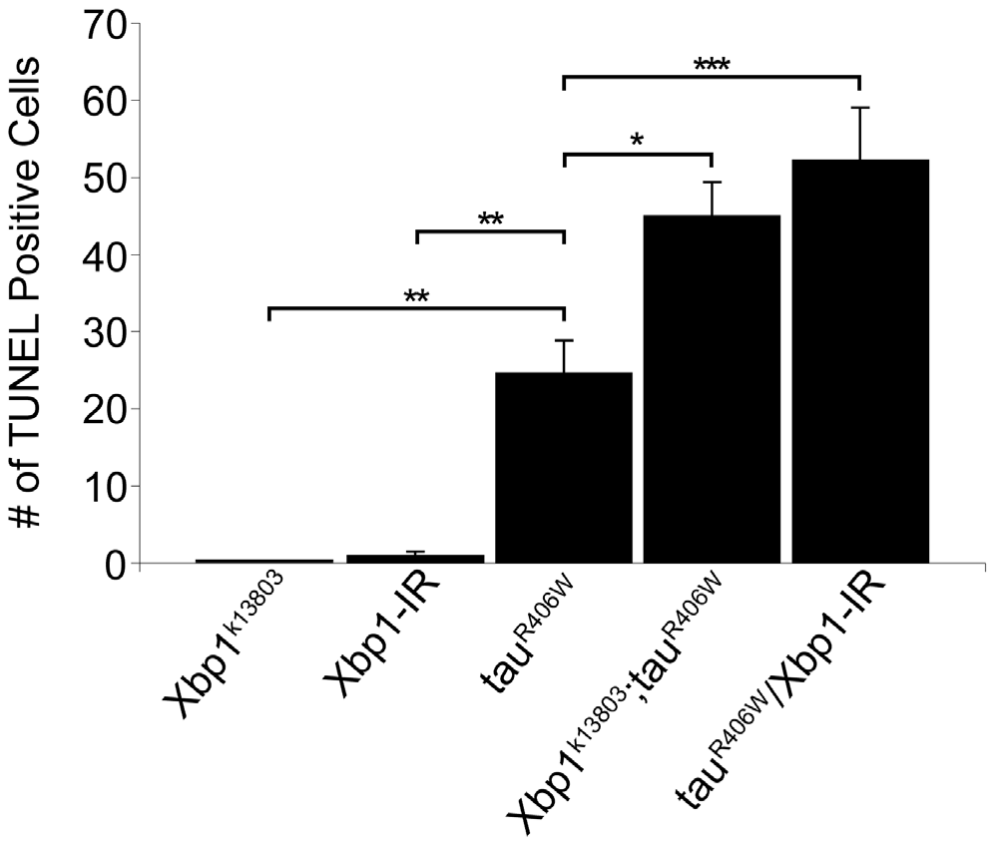
Quantitative analysis of cell death using TUNEL staining. A significant increase in toxicity in flies with reduced levels of Xbp1 using a loss of function allele (*Xbp1^k13803^/+)* or by expressing RNAi to Xpb1 (Xbp1-IR) with the panneural *elav-GAL4* driver. *P<0.05, **P<0.01, *** P<0.001, one-way ANOVA with Tukey test for multiple comparisons. Flies are 10 days old.

To determine whether activation of the UPR influences neurotoxicity, or is merely a marker of cellular toxicity in our model, we determined if manipulation of the UPR modifies tau toxicity. To decrease function of the UPR we utilized a loss of function Xbp1 allele (*Xbp1^k13803^*). *Drosophila* homozygous for the *Xbp1^k13808^* allele have severely reduced Xbp1 transcript levels, exhibit growth retardation and die before reaching the pupal stage [22]. To assess apoptosis in adult fly brains a less severe manipulation of Xbp1 levels was required so we examined *Drosophila Xbp1^k13808^* heterozygotes. Although heterozygosity for *Xbp1^k13808^* itself does not cause apoptosis, quantification of the number of TUNEL-positive cells in 10-day-old fly brains revealed that reducing Xbp1 function with one copy of *Xbp1^k13808^* significantly increased apoptosis mediated by expression of tau (tau^R406W^ = 24.6±4.2, Xbp1^k13808^/+; tau^R406W^ = 44.9±4.4, p<0.05; Figure 3). Thus Xbp1 protects against the toxicity caused by expression of tau. We confirmed these results using an RNAi against Xbp1 (Xbp1-IR). Although expression of Xbp1-IR alone resulted in few TUNEL positive cells (0.8±0.7), its co-expression with tau more than doubled the number of TUNEL positive cells compared to expression of tau alone (tau^R406W^ = 24.6±4.2, tau^R406W^/Xbp1-IR = 52.1±6.9, p<0.001; Figure 3). Enhancement of tau toxicity by reducing Xbp1 levels did not result from increased tau expression because Western blot analysis showed similar levels of tau in control flies (genotype: *elav-GAL4/+;UAS- tau^R406W^/+)* and tau transgenic flies with reduced levels of Xbp1 (genotypes: *elav-GAL4/+;UAS- tau^R406W^/UAS-Xbp1-IR* and *elav-GAL4/+;Xbp1^k13808^/+;UAS- tau^R406W^/+*; Figure S2).

**Figure 4:**
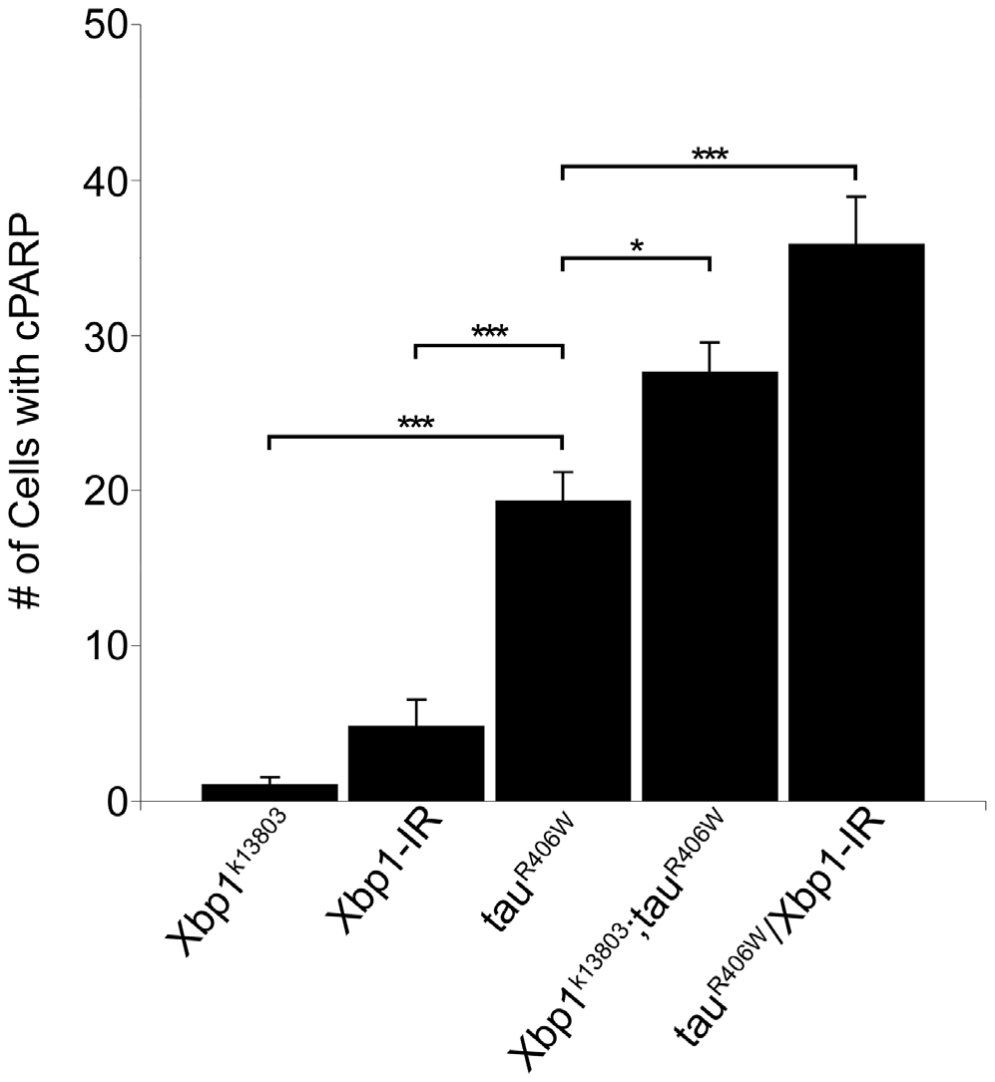
Increased cleaved PARP in flies with reduced levels of Xbp1. Reduction of Xbp1 using a loss of function allele (*Xbp1^k13803^/+)* or RNAi to Xpb1 (Xbp1-IR) with the panneural *elav-GAL4* driver. *P<0.05, **P<0.01, *** P<0.001, one-way ANOVA with Tukey test for multiple comparisons. Flies are 10 days old.

Since cell death is apoptotic in our model and can be decreased by inhibiting caspase activity [23], the increased TUNEL seen in tau transgenic fly brains when an Xbp1 loss of function allele or Xbp1-IR is present suggests that effector caspase activity is increased by reducing Xpb1. To further probe activation of caspase in our system we employed a genetically encoded reporter construct. Transgenic flies have been created that express a caspase substrate, human poly-ADP-ribose polymerase-1 (PARP). Human PARP is cleaved by mammalian caspase-3 and by homologous *Drosophila* effector caspases. Processed PARP can then be recognized by an antibody specific to cleaved human PARP [24]. Human PARP was co-expressed with tau alone and with tau in combination with *Xbp1^k13808^* or Xbp1-IR. An antibody to cleaved PARP was used to detect cleavage and the number of cell bodies with cleaved PARP was quantified. Although heterozygosity for *Xbp1^k13808^* or expression of Xbp1-IR alone resulted in few cells showing evidence of PARP cleavage (*Xbp1^k13803^*/+; PARP = 1.0±0.5 and Xbp1-IR/PARP = 4.8±1.8), decreasing Xbp1 significantly increased the amount of PARP cleavage observed with tau transgenic flies (tau^R406W^, PARP = 19.3±1.9, *Xbp1^k13803^*/+; tau^R406W^, PARP = 27.6±1.9, p<0.05, and Xbp1-IR/tau^R406W^, PARP = 35.8±3.1, p<0.001; Figure 4). Of note, the number of neurons showing UPR activation as measured by the Xbp1-EGFP reporter is similar to the number of TUNEL- and cleaved PARP-positive neurons, consistent with a role for the UPR in tau neurotoxicity.

**Figure 5:**
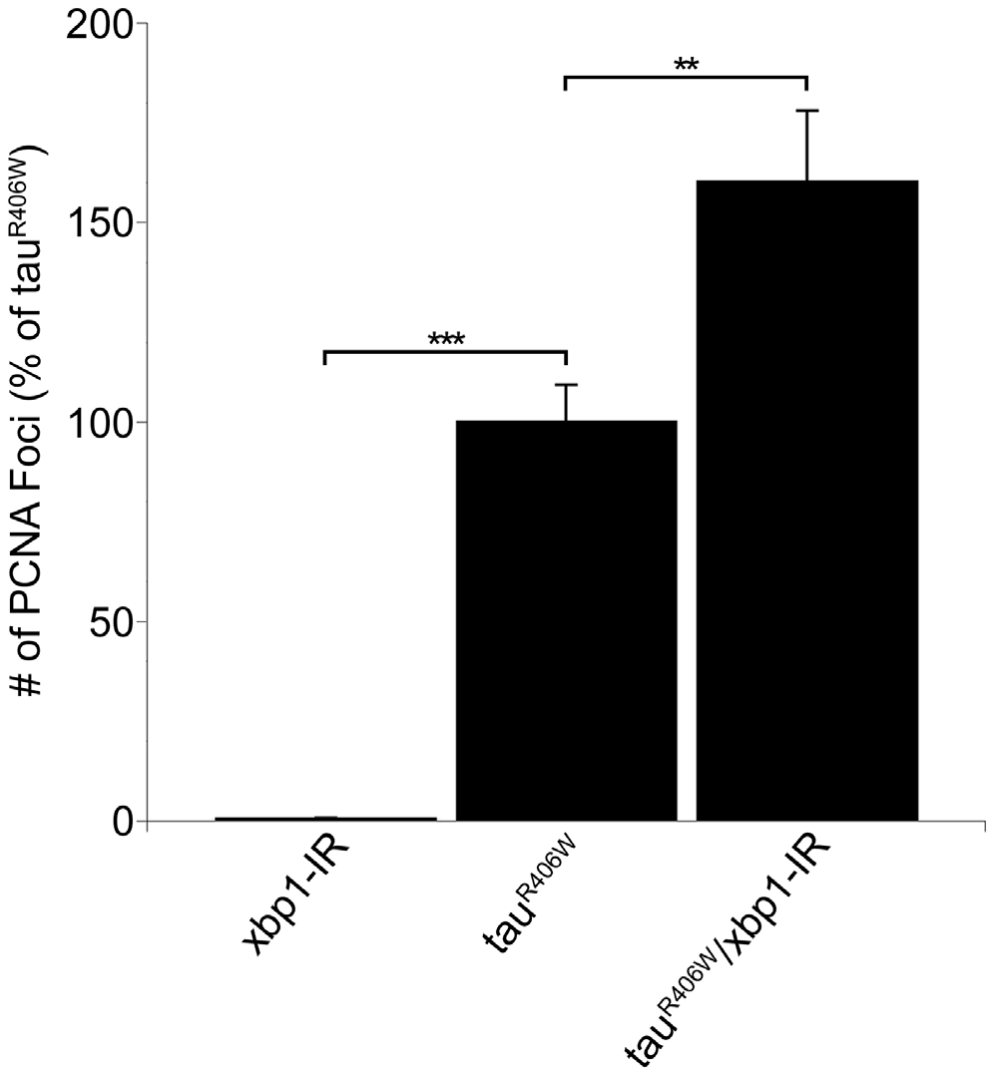
Increased cell cycle activation in flies with reduced levels of Xbp1. PCNA in tau transgenic flies expressing RNAi to Xpb1 (Xbp1-IR) using the panneural *elav-GAL4* driver. **P<0.01, *** P<0.001, one-way ANOVA with Tukey test for multiple comparisons. Flies are 10 days old.

We have previously demonstrated that cell cycle activation causes neurodegeneration in our *Drosophila* tauopathy model [20]. To determine if reducing Xbp1 promotes tau-mediated neurodegeneration via cell cycle activation, fly brains were immunostained with an antibody against the proliferating cell nuclear antigen (PCNA), a marker of cell cycle activation [20], and the number of PCNA positive foci were counted. Although aberrant PCNA expression is minimal with Xbp1-IR alone (0.2±0.2% of tau^R406W^), Xbp1-IR greatly increases the aberrant PCNA expression seen in tau brains (160.2±17.7% of tau^R406W^, p<0.01; Figure 5).

## Discussion

The unfolded protein response has previously been implicated in neurodegenerative diseases. Retinitis pigmentosa is a common form of inherited neurodegeneration often caused by mutations in the photosensitive pigment rhodopsin. Rhodopsin is a large transmembrane protein made at high levels in photoreceptors. Mutations in rhodopsin cause misfolding of the protein within the ER, and significant activation of the UPR [22]. Activation of the UPR is most likely a protective response in retinitis pigmentosa because activating the UPR in *Drosophila* models of rhodopsin misfolding and retinal degeneration protects from photoreceptor loss [22], [25], [26].

Attention has also focused on a possible role for ER stress in Alzheimer's disease. As mentioned above, Aβ peptides can activate the UPR [10], [11]. In addition, altered presenilin function may influence the UPR. Presenilins are multipass transmembrane proteins that reside in the ER. In Alzheimer's disease studies, most attention has focused on the role of presenilins as key components of the γ-secretase complex, which is responsible for cleaving Aβ peptides from the APP precursor [27]. However, presenilins also appear to function as channels that allow release of calcium from the ER lumen. Mutations that predispose to familial Alzheimer's disease impair channel function, leading to increased ER calcium levels [28]. Since increased calcium concentrations within the ER can sensitize to ER stress [29], it is possible that mutations or epigenetic factors that alter presenilin function in Alzheimer's disease could promote neuronal death via ER-related mechanisms.

Less attention has been given to a role for ER stress in controlling toxicity of misfolded cytosolic proteins. Here we show that the UPR is induced in neurons by expression of human tau in a *Drosophila* model of tauopathy (Figures 1 and 2). Our findings contrast somewhat with those of Ryoo et al. [22] who saw only modest induction of the Xbp1-EGFP reporter in the retinas of flies expressing the FTDP-17 associated mutant tau^R406W^. In the current study we examine tau neurotoxity in the context of aging postmitotic neurons of the adult brain. Since evidence exist for impairment of the ER stress response with aging [30], activation of the UPR may be a more important response in aging postmitotic neurons, although we have previously demonstrated good concordance of most genetic modifiers in retinal and brain models of human neurodegenerative diseases in *Drosophila* [31]. Alternatively, it seems plausible that the significant ER stress caused by misfolding of the abundant photoreceptor pigment rhodopsin simply induces greater UPR than the soluble cytosolic protein tau. Nonetheless, the UPR induction we observe in our model is functionally important because we can significantly increase neurotoxicity of tau when we reduce function of the ER stress system by reducing levels of Xbp1 (Figures 3 and 4).

To address the mechanism by which tau might activate the UPR we first determined if activation of the UPR was related to phosphorylation of tau by using a phosphomimic version of tau with substantially increased neurotoxicity [16]–[20]. Phosphorylation of tau on proline directed sites (so called S/P and T/P sites) has been strongly implicated in the toxicity of tau in Alzheimer's disease [1], and in *Drosophila* models of tauopathy as well [14], [16]–[20]. We found that pseudophosphorylation of tau at 14 S/P and T/P sites significantly increased UPR activation, suggesting that phosphorylation of tau occurs before, and promotes, UPR activation. These findings are consistent with results from autopsy studies that have associated activation of the UPR with phosphorylation of tau at the AT8 epitope in Alzheimer's disease patients [12].

We also probed the mechanism by which reduced ER stress response leads to increased neurodegeneration. We have previously shown that tau-induced cell cycle reentry mediates neuronal apoptosis in the fly tauopathy model [20], a result that has been corroborated in rodent models model of tauopathy [32], [33]. To test whether reducing the ER stress response enhanced tau-induced neurodegeneration through abnormal cell-cycle activation, we examined the levels of proliferating cell nuclear antigen (PCNA), an S-phase cell-cycle marker abnormally upregulated in brains of Alzheimer's disease patients [34] and tau-expressing flies. Since reducing levels of Xbp1 increased PCNA levels (Figure 5) as well as markers of cell toxicity (Figures 3 and 4), reducing function of the ER stress response most likely promotes tau neurotoxicity through abnormal cell cycle activation.

Taken together, the results we present in the current study support a role for the cytosolic protein tau in activating the UPR. Our findings are consistent with studies from human postmortem tissue in which activation of the UPR was seen in two cases of tauopathies with pathological features of progressive supranuclear palsy and corticobasal degeneration, conditions not associated with Aβ deposition [35]. ER stress might thus represent a point of convergence between the dual pathologies of Aβ and tau in Alzheimer's disease. Our genetic data suggests that activation of the UPR may play a neuroprotective role in tauopathies because reducing Xbp1 levels exacerbates cellular toxicity. Activation of the UPR and evaluation of the effects on tau neurotoxicity represents an additional approach to exploring the role of the UPR in tau neurotoxicity. Our findings are in contrast to those obtained in a model of amyotrophic lateral sclerosis caused by expression of a distinct cytosolic misfolded protein, mutant superoxide dismutase (SOD). In a murine model of amyotrophic lateral sclerosis based on expression of G93A mutant SOD, removing Xbp1 protected from neurotoxicity, presumably through activation of a neuroprotective autophagic response [36]. In contrast, our findings suggest that activating, rather than inhibiting, the ER stress response may be a potential therapeutic avenue in Alzheimer's disease and related tauopathies.

## Materials and Methods

### Fly Stocks, Genetics

All fly crosses and experiments were performed at 25°C. All flies were analyzed at 10 days of age. The *elav-GAL4* driver and *Hsc70-3^G0407^* were obtained from the Bloomington *Drosophila* Stock Center. The Xbp1-EGFP reporter and *Xbp1^k13803^* were the kindly provided by H.D. Ryoo. The human PARP transgenic flies are described in [24]. *UAS-Xbp1-IR* was obtained from the Vienna Drosophila RNAi Center.

### Sectioning, Immunostaining, and Histology

Adult flies were fixed in formalin at 10 days of age and embedded in paraffin. Serial frontal 4 µm sections including the entire brain were prepared. Antigen retrieval was performed by microwaving in sodium citrate buffer, pH 6.0. Immunostaining was performed with an avidin-biotin-peroxidase complex (ABC) method (Vector Laboratories) or with secondary antibodies coupled to Alexa Fluor 488 or Alexa Fluor 555. Primary antibodies included anti-elav (Developmental Studies Hybridoma Bank), anti-repo (Developmental Studies Hybridoma Bank), anti-PCNA (DAKO), anti-GFP (NeuroMab), anti-PARP p85 fragment (Promega), and anti-ß-galactosidase (Promega). For quantitative analysis, the number of GFP-immunoreactive nuclei (Xbp1-EGFP reporter), ß-galactosidase-immunoreactive nuclei (*Hsc70-3-lacZ* reporter), cleaved PARP-positive cells, or PCNA-positive foci were counted in the entire brain. At least six brains were analyzed per genotype. Statistical significance was established by one-way ANOVA with a Tukey test for multiple comparisons.

### TUNEL Staining

Apoptosis was detected with the TUNEL assay with a commercially available kit (TdT FragEl, Oncogene). Neurodegeneration was quantified by counting the number of TUNEL-positive cells per brain in consecutive frontal sections between the ellipsoid body anteriorly and the mushroom body posteriorly. At least six brains were examined per time point for each genotype. Statistical significance was established by one-way ANOVA with a Tukey test for multiple comparisons.

### Western Blots

Adult *Drosophila* heads were homogenized in 2X Laemmli buffer (Sigma-Aldrich). Samples were boiled for 10 minutes, briefly centrifuged and subjected to SDS-PAGE in 10% separating gels (Cambrex). Proteins were transferred to nitrocellulose membranes (Bio-Rad), blocked in 2% milk in phosphate-buffered saline with 0.05% Tween-20, and immunoblotted using a phosphorylation-independent rabbit polyclonal anti-tau antibody (1∶10^6^; Dako). Ponceau S staining was used to evaluate protein loading and transfer in all Western blots. Western blots were also reprobed with an antibody recognizing actin (1∶50,000; Sigma-Aldrich) to illustrate equivalent protein loading.

## Supporting Information

Figure S1 Western blot of total tau levels using a phosphorylation-independent polyclonal anti-tau antibody (Dako) reveals equivalent levels of tau expression in flies expressing tauWT compared to tauR406W, and slightly less expression of tauE14 compared to tauWT, despite increased toxicity in tauE14 transgenic flies (Figure 2). Control is elav-GAL4/+. The blots were reprobed for actin as a loading control (lower panels). Flies are 10 days old.
(0.67 MB TIF)

Figure S2 Western blot of total tau levels reveals equivalent levels of tau expression in flies heterozygous for an Xbp1 loss of function allele (Xbp1k13803) and in flies expressing RNAi to Xpb1 (Xbp1-IR). Control is elav-GAL4/+. The blot was reprobed for actin as a loading control (lower panel). Flies are 10 days old.
(1.09 MB TIF)
