# Supplementary figures and images for: The Unfolded Protein Response Protects from Tau Neurotoxicity In Vivo
Source: PLoS One. 2010 Sep 29;5(9):e13084. doi: 10.1371/journal.pone.0013084 (PMC2947516; doi:10.1371/journal.pone.0013084)

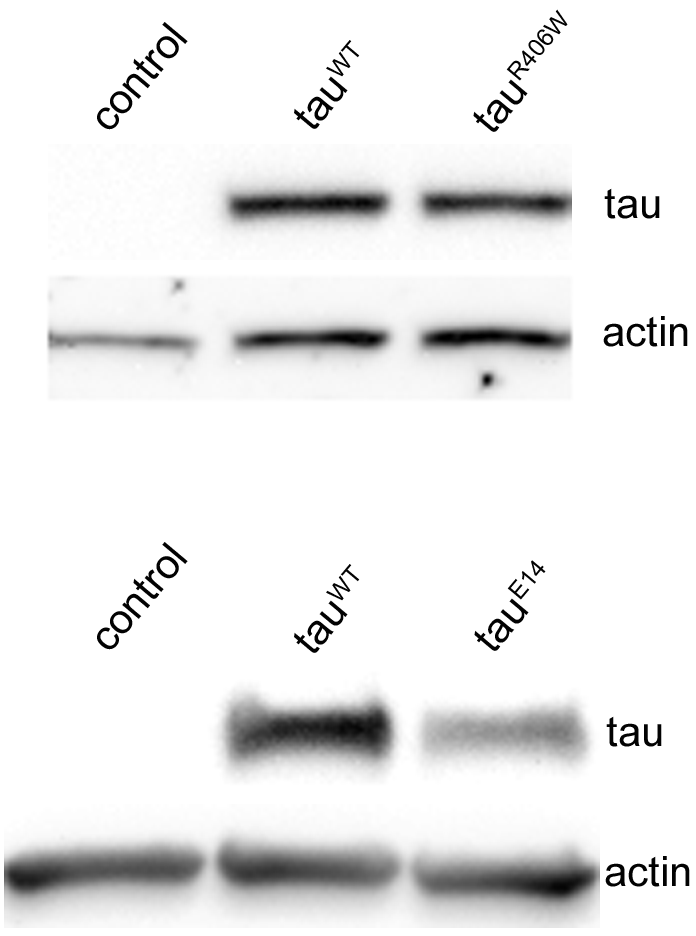

Supplement: Figure S1 — Western blot of total tau levels using a phosphorylation-independent polyclonal anti-tau antibody (Dako) reveals equivalent levels of tau expression in flies expressing tauWT compared to tauR406W, and slightly less expression of tauE14 compared to tauWT, despite increased toxicity in tauE14 transgenic flies (Figure 2). Control is elav-GAL4/+. The blots were reprobed for actin as a loading control (lower panels). Flies are 10 days old. (0.67 MB TIF) [file pone.0013084.s001.tif]

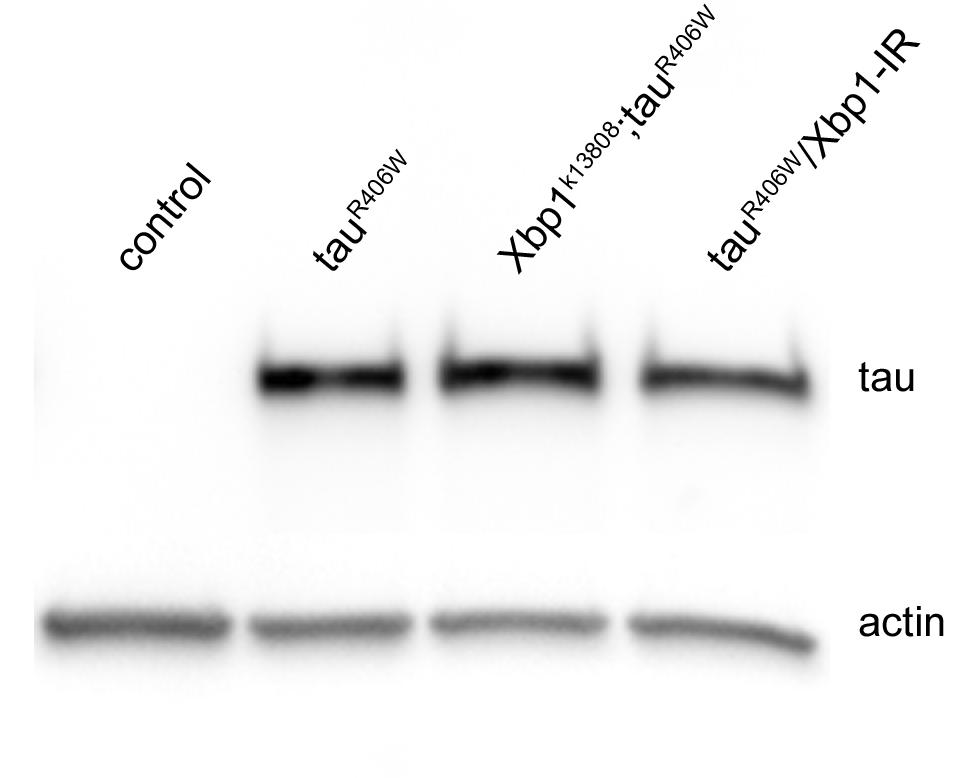

Supplement: Figure S2 — Western blot of total tau levels reveals equivalent levels of tau expression in flies heterozygous for an Xbp1 loss of function allele (Xbp1k13803) and in flies expressing RNAi to Xpb1 (Xbp1-IR). Control is elav-GAL4/+. The blot was reprobed for actin as a loading control (lower panel). Flies are 10 days old. (1.09 MB TIF) [file pone.0013084.s002.tif]
